# A construction and comprehensive analysis of the immune-related core ceRNA network and infiltrating immune cells in peripheral arterial occlusive disease

**DOI:** 10.3389/fgene.2022.951537

**Published:** 2022-09-15

**Authors:** Zhiyong Chen, Jiahui Xu, Binshan Zha, Jun Li, Yongxiang Li, Huan Ouyang

**Affiliations:** ^1^ Department of Vascular and Thyroid Surgery, Department of General Surgery, First Affiliated Hospital of Anhui Medical University, Hefei, China; ^2^ Department of General Medicine, First Affiliated Hospital of Anhui Medical University, Hefei, China; ^3^ Department of General Surgery, First Affiliated Hospital of Anhui Medical University, Hefei, China

**Keywords:** peripheral arterial occlusive disease, PAOD, ceRNA, immune cell infiltration, atherosclerosis

## Abstract

**Background:** Peripheral arterial occlusive disease (PAOD) is a peripheral artery disorder that increases with age and often leads to an elevated risk of cardiovascular events. The purposes of this study were to explore the underlying competing endogenous RNA (ceRNA)-related mechanism of PAOD and identify the corresponding immune cell infiltration patterns.

**Methods:** An available gene expression profile (GSE57691 datasets) was downloaded from the GEO database. Differentially expressed (DE) mRNAs and lncRNAs were screened between 9 PAOD and 10 control samples. Then, the lncRNA-miRNA-mRNA ceRNA network was constructed on the basis of the interactions generated from the miRcode, TargetScan, miRDB, and miRTarBase databases. The functional enrichment and protein–protein interaction analyses of mRNAs in the ceRNA network were performed. Immune-related core mRNAs were screened out through the Venn method. The compositional patterns of the 22 types of immune cell fraction in PAOD were estimated through the CIBERSORT algorithm. The final ceRNA network and immune infiltration were validated using clinical tissue samples. Finally, the correlation between immune cells and mRNAs in the final ceRNA network was analyzed.

**Results:** Totally, 67 DE_lncRNAs and 1197 DE_mRNAs were identified, of which 130 DE_mRNAs (91 downregulated and 39 upregulated) were lncRNA-related. The gene ontology enrichment analysis showed that those down- and upregulated genes were involved in dephosphorylation and regulation of translation, respectively. The final immune-related core ceRNA network included one lncRNA (*LINC00221*), two miRNAs (*miR-17-5p* and *miR-20b-5p*), and one mRNA (*CREB1*). Meanwhile, we found that monocytes and M1 macrophages were the main immune cell subpopulations in PAOD. After verification, these predictions were consistent with experimental results. Moreover, *CREB1* was positively correlated with naive B cells (*R* = 0.55, *p* = 0.035) and monocytes (*R* = 0.52, *p* = 0.049) and negatively correlated with M1 macrophages (*R* = −0.72, *p* = 0.004), resting mast cells (*R* = −0.66, *p* = 0.009), memory B cells (*R* = −0.55, *p* = 0.035), and plasma cells (*R* = −0.52, *p* = 0.047).

**Conclusion:** In general, we proposed that the immune-related core ceRNA network (*LINC00221*, *miR-17-5p*, *miR-20b-5p*, and *CREB1*) and infiltrating immune cells (monocytes and M1 macrophages) could help further explore the molecular mechanisms of PAOD.

## Introduction

Peripheral arterial occlusive disease (PAOD) is an atherosclerotic condition involving non-cardiac and non-cerebral arteries. Nowadays, it has developed into a widespread disease with more than 200 million people affected worldwide and has become the third most common cause of death from cardiovascular disease (Fowkes et al., 2013). The importance of it is growing by virtue of its increasing incidence. Patients with PAOD often suffer from chronic limb ischemia that results in intermittent claudication, resting pain, disability, and even death. As PAOD is one common manifestation of systemic atherosclerosis, it is essential to study peripheral atherosclerosis for exploring the potential pathogenesis and progression of PAOD and effective therapeutic targets.

In recent years, more and more studies have shown that atherosclerosis, as a chronic inflammatory disease, is significantly associated with the infiltration of immune cells such as neutrophils, macrophages, T cells, and B cells into the inner layer of the vessel wall (Hansson and Hermansson, 2011; Baptista et al., 2018). In atherosclerosis, hypercholesterolemia leads to the accumulation of plasma low-density lipoprotein (LDL) in the artery wall, which stimulates and recruits monocytes and elicits local inflammation. Then, monocytes are infiltrated to differentiate locally into macrophages, and the lipid metabolic disorders and efferocytosis of macrophages are reduced, leading to irreversible inflammation (Tabas and Bornfeldt, 2016). Macrophages polarized to M1 and M2 exert pro-inflammatory and anti-inflammatory effects, respectively (Moore and Tabas, 2011). T cells account for about 40% of the total number of immune cells in human atherosclerotic lesions. Among them, regulatory T (Treg) cells produce transforming growth factor β, which inhibits the proliferation of T-helper type 1 (Th1) and T-helper type 17 (Th17) cells (Fantini et al., 2006). Th1 cells and natural killer (NK) cells secrete pro-inflammatory factors, which destroy collagen fibers and promote the transformation of atherosclerotic plaques to vulnerable phenotypes (Konkel et al., 2017). Th17 cells are a subtype of T cells, which can promote the formation of thick collagen fibers and contribute to the stability of plaques (Gisterå et al., 2013). Dendritic cells (DCs) can make the innate and adaptive immune responses to act as important modulators in atherosclerosis (Cybulsky et al., 2016). Several B-lymphocyte subsets contribute to the inflammatory process of atherosclerosis through cellular and humoral responses (Tsiantoulas et al., 2014). However, in PAOD, the landscape of immune cell infiltration has not been fully elucidated. Moreover, the relationship between immune-related genes and immune cells in PAOD is largely unknown.

Noncoding RNAs (ncRNAs) regulate gene expression at transcriptional and posttranscriptional levels without coding proteins in the transcriptome, including microRNAs (miRNAs) and long noncoding RNAs (lncRNAs). miRNAs are a class of highly conserved single-stranded noncoding small RNAs, which contain approximately 19–25 nucleotides and have post-transcriptional regulatory activity. lncRNAs are defined as a type of ncRNAs that are longer than 200 nucleotides in length, with multilevel regulatory functions in gene expression, such as transcription, translation, and epigenetics. Accumulating evidence has shown that functional ncRNAs play an important role in the pathogenesis and progression of many diseases, such as cancer, digestive system diseases, and cardiovascular diseases. In recent years, a competing endogenous RNA (ceRNA) network hypothesis has been proposed (Salmena et al., 2011). In the ceRNA network, lncRNAs can serve as endogenous molecular sponges for miRNAs to regulate the expression of messenger RNAs (mRNAs) indirectly. In this way, the function of ncRNAs can be linked to the function of mRNAs that encode proteins. Given their complexity, the dysregulation of the lncRNA-miRNA-mRNA network is closely related to the pathogenesis and progression of many human diseases, such as cardiovascular diseases. For instance, Ye et al. (2019) found that lncRNA *MIAT* upregulates CD47 expression by sponging *miR-149-5p* and inhibits efferocytosis in advanced atherosclerosis. Yang et al. (2021) discovered that the lncRNA *XIST* serves as a ceRNA and promotes atherosclerosis by increasing *miR-599*-mediated expression of *TLR4*. Nevertheless, few data-based studies have been conducted to analyze the relationship between the immune-related ceRNA regulatory network and infiltrating immune cells in PAOD.

In this study, we compared differentially expressed (DE) mRNAs and lncRNAs between 9 PAOD and 10 control samples downloaded from the Gene Expression Omnibus (GEO) database. Then, the target miRNAs of DE_lncRNAs and DE_mRNAs of target miRNAs were predicted. Subsequently, protein–protein interaction (PPI) analysis among the predicted DE_mRNAs was conducted, and hub DE_mRNAs were identified by the Cytoscape’s cytoHubba plugin. The overlapping genes between the hub DE_mRNAs and immune-related genes were identified as core mRNAs to construct the potential immune-related core ceRNA regulatory network of PAOD. Meanwhile, the CIBERSORT method was used to analyze the different patterns of immune cell infiltration in PAOD. The immune-related core ceRNA network and immune infiltration were validated using clinical tissue samples. Finally, we investigated and visualized the correlation between the core mRNAs and infiltrating immune cells in an effort to better understand the molecular immune mechanism during the progression of PAOD.

## Materials and methods

### Data acquisition

In this study, the microarray dataset GSE57691 (Biros et al., 2015) that assessed the relative gene expression in human abdominal aortic aneurysm (AAA) and PAOD, and GSE137580 that studied the global miRNAs in atherosclerotic models that oxidative LDL treated human aortic endothelial cells (HAEC) were obtained from the Gene Expression Omnibus database (GEO, http://www.ncbi.nlm.nih.gov/geo) (Barrett et al., 2013). The specimens of GSE57691 were obtained from 20 patients with small AAA, 29 patients with large AAA, 9 PAOD patients, and 10 organ donors. Then, the data on PAOD and normal artery were picked out for further analysis. The test platforms of GSE57691 and GSE137580 were GPL10558 Illumina HumanHT-12 V4.0 expression beadchip and GPL24741 Agilent-070156 Human_miRNA_V21.0_Microarray 046064 (gene name version), respectively. The GSE137580 data were used to validate the relative expression level of miRNAs in the potential ceRNA network. Additionally, immune-related genes were downloaded from the Immunology Database and Analysis Portal (ImmPort) database (http://www.immport.org/) (Bhattacharya et al., 2014).

### Differential expression analysis

First, the probe sets were converted into corresponding gene symbols according to the platform profile with annotation information. If multiple probe sets correspond to the same gene, their mean value was calculated by R software (version 4.1.0). Then, based on the gene annotation information included in the ENSEMBL database (https://asia.ensembl.org/Homo_sapiens/Info/Index), the expression profile dataset was divided into lncRNA and mRNA groups. The linear models for the microarray data (LIMMA) package of R software were utilized to normalize raw data and perform DE_RNA analysis between PAOD and normal artery groups (Ritchie et al., 2015). *p*-values were adjusted by the Benjamini–Hochberg (BH) false discovery rate (FDR) method (Glickman et al., 2014). The cut-off value of DE_RNAs was set as adj. *p*-value < 0.01 and |fold change (FC)| > 1.5 (Meng et al., 2019). The heatmap of the DE_lncRNAs and the volcano plot of all RNAs were constructed for data visualization by the “pheatmap” (https://CRAN.R-project.org/package=pheatmap) and “ggplot2” packages in R software, respectively (Ginestet, 2011).

### Prediction of lncRNA-miRNA-mRNA interactions

The highly conserved miRNA families of the miRcode database (http://www.mircode.org/) were applied to predict interactions between DE_lncRNAs and potential miRNAs (Jeggari et al., 2012). Subsequently, the TargetScan (http://www.targetscan.org/) (Agarwal et al., 2015), miRDB (http://www.mirdb.org/) (Chen and Wang, 2020), and miRTarBase (http://mirtarbase.mbc.nctu.edu.tw/) (Huang et al., 2020) databases were utilized to forecast miRNA–mRNA pairs. Only those genes that concurrently existed in all three databases were considered as candidate targets of mRNAs for further analysis.

### Venn method

The Venn method was employed to analyze overlapping genes. Intersections between DE_lncRNA predicted mRNAs and DE_mRNAs, as well as immune-related genes and lncRNA-related DE_mRNAs (the intersections between DE_lncRNA predicted mRNAs and DE_mRNAs), were calculated using an online tool. (http://bioinformatics.psb.ugent.be/webtools/Venn/). Moreover, the tool was also used to identify the hub genes.

### Functional enrichment and protein–protein interaction (PPI) analysis

First, the lncRNA-related DE_mRNAs were divided into expression upregulated and downregulated groups. Then, the “clusterProfiler” package (Yu et al., 2012) in R software was used to perform gene ontology (GO) (Gene Ontology Consortium, 2006) and Kyoto Encyclopedia of Genes and Genomes (KEGG) (Kanehisa et al., 2010) enrichment analyses. GO enrichment analysis included three categories: cellular component (CC), molecular function (MF), and biological process (BP). The “ggplot2” package in R software was utilized to draw the bubble charts for visualization of the results of the GO and KEGG enrichment analyses. *p*-value < 0.05 was considered significantly enriched when screening. Subsequently, the online Search Tool for the Retrieval of Interacting Genes (STRING, https://string-db.org/) database (Szklarczyk et al., 2019) was used to determine the relationship between the lncRNA-related DE_mRNAs. The minimum required interaction score was 0.7, and the disconnected nodes in the network were hidden. Then, Cytoscape software (version 3.8.2) was used to develop the PPI network. Furthermore, the Maximal Clique Centrality (MCC), Degree, and Maximum Neighborhood Component (MNC) algorithms in the cytoHubba plugin were used to screen out the top 10 mRNAs in the PPI network. The overlapping genes obtained by the three different aforementioned algorithms were identified as hub genes. Finally, we identified the overlapping genes between the hub genes before and the immune-related genes as immune-related core genes.

### Construction of the immune-related core ceRNA network

First, the interactions between lncRNAs, miRNAs, and immune-related core mRNAs were confirmed, as described in the aforementioned item. Second, the immune-related lncRNA-miRNA-mRNA ceRNA network was developed, and the “ggalluvial” package (http://corybrunson.github.io/ggalluvial/) in R software was used to draw a Sankey diagram for data visualization. Subsequently, a correlation analysis between lncRNA and immune-related core genes in the ceRNA network was performed by the “LIMMA” package in R software. Ultimately, the relative expression levels of miRNAs in immune-related core ceRNA were validated in the GSE137580 dataset and visualized by GraphPad Prism (version 7.0). *p*-value < 0.05 was considered statistically significant.

### Estimation of immune cell infiltration

CIBERSORT (https://cibersort.stanford.edu/) is a versatile analytical tool that uses gene expression data to quantify the cell fractions from complex tissues and has been confirmed by flow cytometry (Newman et al., 2015). To analyze the proportion of 22 infiltrating immune cells in atherosclerotic plaques of PAOD patients and normal controls, the mRNA expression data were uploaded to the CIBERSORT platform. Only samples that had a CIBERSORT algorithm output of *p*-value < 0.05 were filtered out, and the immune cell infiltration matrix was obtained for further analysis. Histograms and heatmaps were drawn to show the rate of immune cell infiltration in different samples. Subsequently, the Wilcoxon rank-sum test was performed to assess the differential composition of infiltrating immune cells between PAOD patients and controls. Results were visualized by the “pheatmap” and “vioplot” (https://github.com/TomKellyGenetics/vioplot) packages in R software. Furthermore, Pearson’s correlation analysis was adopted to explore the correlation among 22 immune cell subtypes. A correlation heatmap was drawn by the “corrplot” package (https://github.com/taiyun/corrplot) in R software to visualize the correlation analysis results. Finally, the “ggstatsplot” package (https://github.com/IndrajeetPatil/ggstatsplot) was used to perform correlation analysis on the immune-related core DE_mRNAs and infiltrating immune cells, and the “ggplot2” package was used to visualize the results in R software.

### Real-time quantitative PCR

From June 2021 to February 2022, we recruited five patients with PAOD who underwent femoral endarterectomy and five organ donors who donated normal iliac arteries in the First Affiliated Hospital of Anhui Medical University. All tissue specimens were divided into two parts and frozen in liquid nitrogen immediately when they were isolated. One part of these samples was pretreated, and total RNA was extracted with TRIzol reagent (Invitrogen Life Technologies, United States) for performing real-time quantitative polymerase chain reaction (RT-qPCR). Spectrophotometry was used to measure the purity of RNA. RNAs were reverse transcribed into complementary DNAs using the Bestar qPCR RT Kit (DBI, Germany), following the instructions of the manufacturer. Subsequently, complementary DNA was amplified by RT-qPCR using an Applied Biosystems SYBR Green mix kit (ABI, United States). GAPDH was used as an internal reference for lncRNAs and mRNAs, while U6 was used as a reference for miRNAs. Primer sequences were obtained from PrimerBank and miRprimer2 databases (Supplementary Table S1). The reactions were measured on the ABI 7900HT Real-Time PCR system (ABI, United States), and the 2^−ΔΔCT^ method was used for analysis.

### Hematoxylin and eosin (H&E) and immunofluorescence staining

The other part of those samples was demineralized after fixation in 4% paraformaldehyde and then embedded in paraffin. All samples were cut into 4-μm slices for further staining. H&E staining was performed to assess the atherosclerotic lesions. The protein expression levels of CD68 and iNOS were analyzed by immunofluorescence staining. Antibodies (i.e., CD68 and iNOS) were purchased from Proteintech (Chicago, IL, United States). All procedures were conducted according to the recommendations of the manufacturer. Images were observed with a fluorescence microscope (Leica DMI6000B, Germany) and analyzed by ImageJ software.

### Statistical analysis

The data are presented as the mean ± standard deviation (SD). SPSS (SPSS Inc., Chicago, IL, United States, version: 19.0) was used to conduct statistical analysis. Student’s t-test was used for comparisons between two groups. A *p*-value of less than 0.05 was considered statistically significant. All experiments were performed at least three times.

## Results

### Identification of DE mRNAs and lncRNAs

**FIGURE 1:**
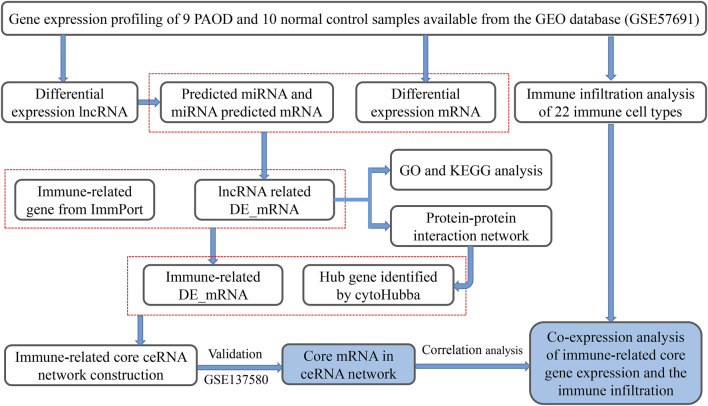
Schematic representation of our analytic process.

**FIGURE 2:**
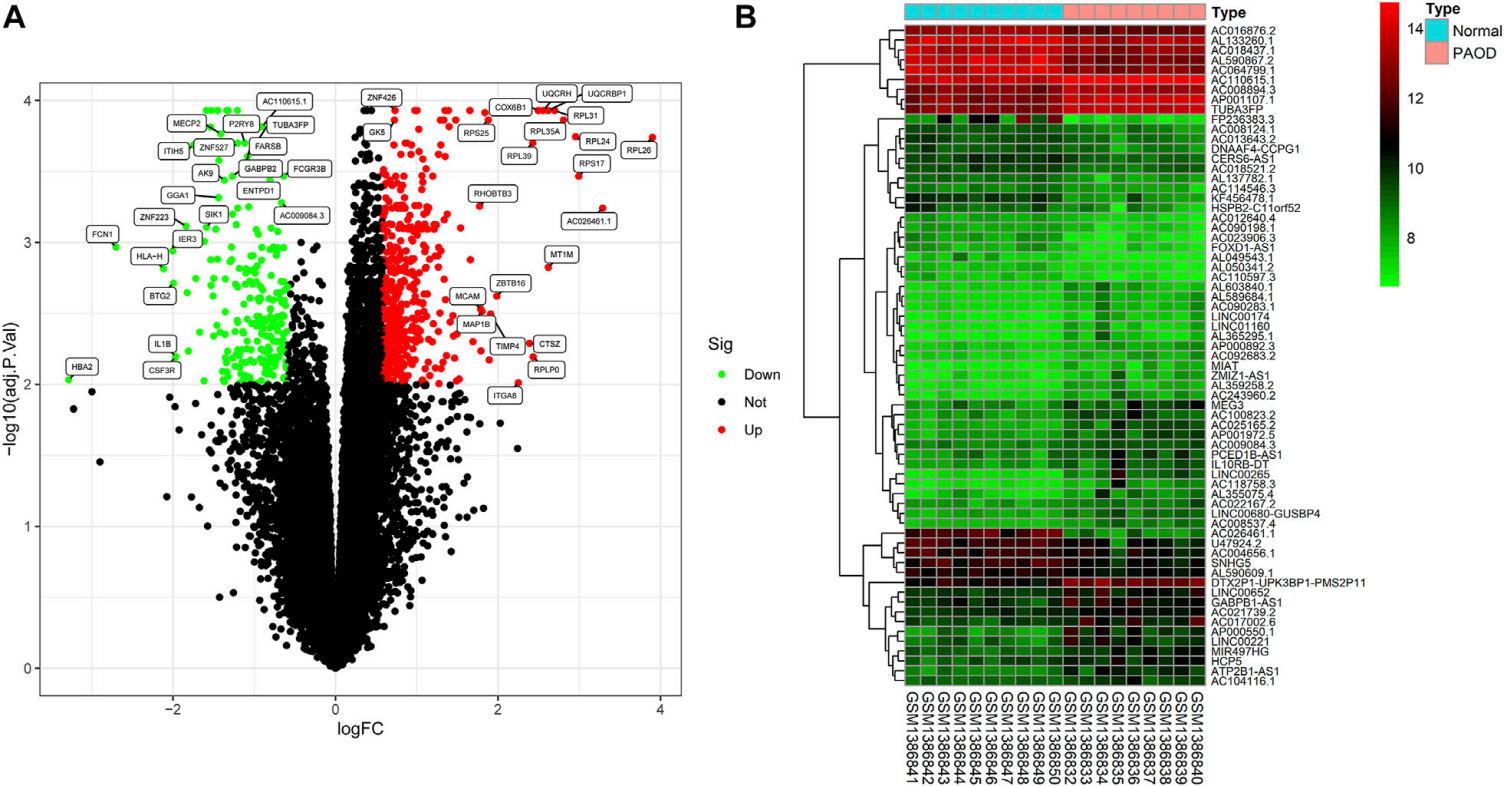
Differentially expressed (DE) mRNAs and lncRNAs in tissues between PAOD patients and normal controls. **(A)** Volcano plot of DE_mRNAs. **(B)** Heatmap of DE_lncRNAs.

In order to clarify the process of this research and make it easier for readers to read, a schematic representation is provided in Figure 1. Raw data were downloaded from the GSE57691 dataset in the GEO database. In total, RNA-seq data from 9 PAOD and 10 normal tissues were analyzed using criteria of |fold change (FC)| > 1.5 and adj. *p*-value < 0.01. A total of 2,142 lncRNAs and 18,219 mRNAs were re-annotated according to the platform profile with annotation information. Moreover, we identified 1264 DE RNAs, including 67 lncRNAs (27 downregulated and 40 upregulated) and 1197 mRNAs (752 down-regulated and 445 up-regulated) meeting the thresholds. Then, the corresponding volcano plot and heatmap of DE_lncRNAs are shown in Figure 2.

### Functional enrichment analysis of lncRNA-related DE_mRNAs

**FIGURE 3:**
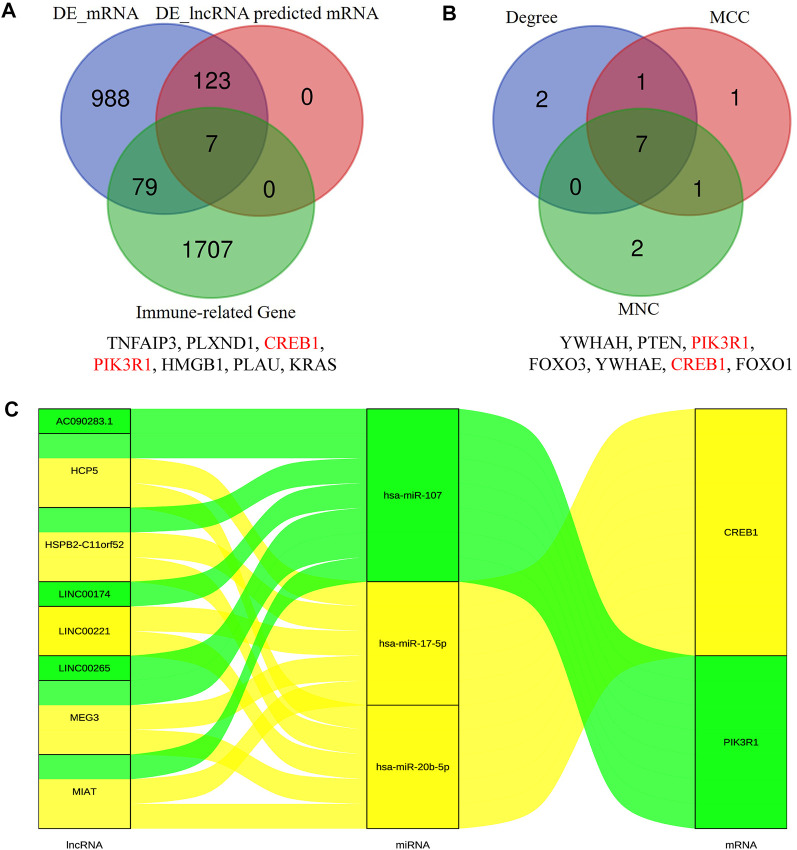
Identification of lncRNA-related DE-mRNAs **(A)** and hub genes **(B)**. The details of intersection in the Venn diagram are listed as follows. Construction of the immune-related core ceRNA network in PAOD **(C)**. MCC, maximal clique centrality; MNC, maximum neighborhood component.

**FIGURE 4:**
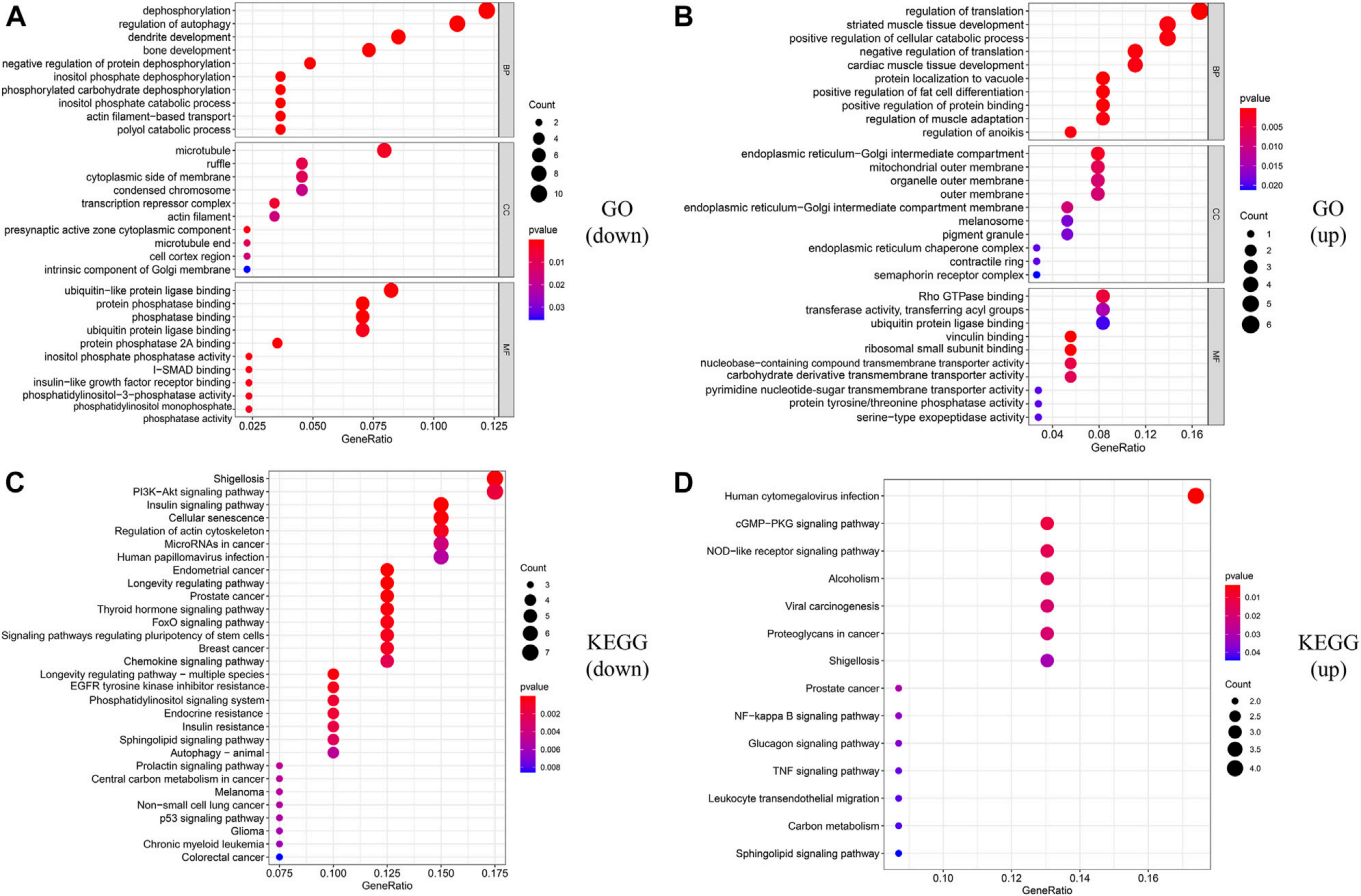
Functional enrichment analysis of lncRNA-related DE_mRNAs. **(A**,**B)** GO enrichment analysis of downregulated and upregulated lncRNA-related DE-mRNAs. **(C**,**D)** KEGG enrichment analysis of downregulated and upregulated lncRNA-related DE-mRNAs.

In order to construct the ceRNA network, DE_lncRNAs were further analyzed. The miRcode database was employed to predict potential DE_lncRNA-targeted miRNAs. Then, potential miRNA–mRNA pairs were analyzed using the miRTarBase, TargetScan, and miRDB databases. A total of 34 miRNAs were identified as DE_lncRNA-predicted miRNAs, and 130 mRNAs (91 downregulated and 39 upregulated) were identified as DE_lncRNA-predicted mRNAs. Subsequently, the Venn method was used to analyze the overlapping genes between DE_mRNAs and DE_lncRNA-predicted mRNAs. As shown in Figure 3A, the intersection contains all of the DE_lncRNA-predicted mRNAs. These mRNAs were also called lncRNA-related DE_mRNAs. To determine the potential mechanisms of lncRNA-related DE_mRNAs, these mRNAs were divided into down- and upregulated groups for further GO and KEGG enrichment analyses (Figures 4A–D). A biological process analysis showed that mRNAs in the downregulated group were significantly enriched in dephosphorylation, regulation of autophagy, and inositol phosphate catabolic processes, while mRNAs in the upregulated group were mostly enriched in regulation of translation, positive regulation of the cellular catabolic process, and positive regulation of fat cell differentiation. A cellular component analysis showed that mRNAs in the downregulated group were significantly enriched in microtubules, while mRNAs in the upregulated group were mostly enriched in the endoplasmic reticulum–Golgi intermediate compartment. A molecular function analysis showed that mRNAs in the downregulated group were significantly enriched in ubiquitin-like protein ligase binding and phosphatase binding, while mRNAs in the upregulated group were mostly enriched in Rho GTPase binding and vinculin binding. The KEGG pathway enrichment analysis showed mRNAs in the downregulated group were significantly enriched in the PI3K-Akt signaling pathway, cellular senescence, and regulation of the actin cytoskeleton. However, mRNAs in the upregulated group were mostly enriched in human cytomegalovirus infection and the cGMP-PKG signaling pathway.

### PPI network construction and hub gene identification

**FIGURE 5:**
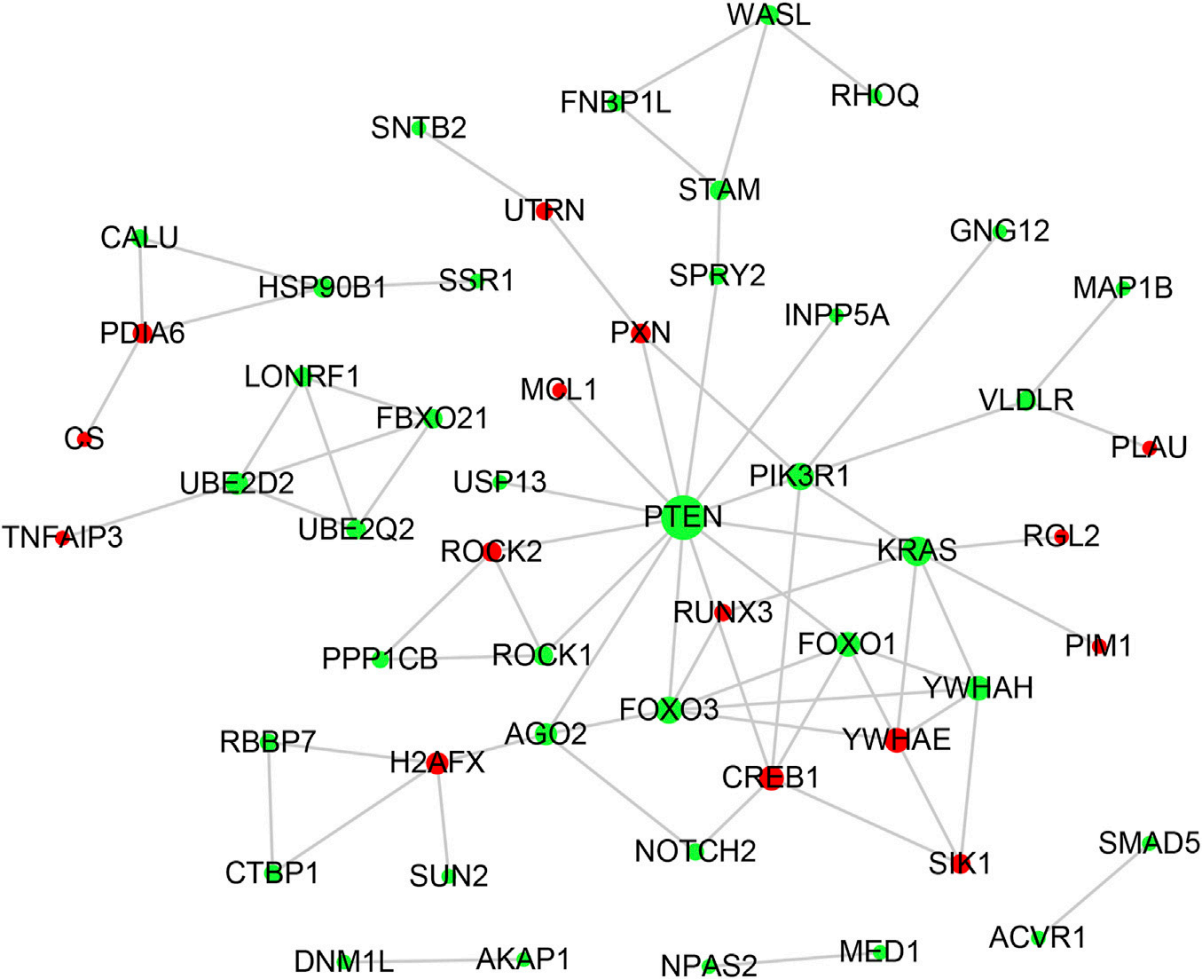
PPI network analysis. The PPI network consisting of 130 nodes and 61 edges was visualized in Cytoscape. Red and green notes symbolize the upregulated and downregulated genes. The size of each node is positively correlated with its degree value.

The PPI network of lncRNA-related DE_mRNAs containing 130 nodes and 61 edges was constructed based on the STRING online database and visualized by Cytoscape software (Figure 5). Subsequently, Cytoscape’s plugin cytoHubba was used to identify the top 10 genes based on three commonly used classification methods (MCC, Degree, and MNC) (Supplementary Table S1). By overlapping these genes, seven hub genes (*YWHAH*, *PTEN*, *PIK3R1*, *FOXO3*, *YWHAE*, *CREB1*, and *FOXO1*) were consequently identified, as shown in Figure 3B.

### Construction of the immune-related core ceRNA network

**FIGURE 6:**
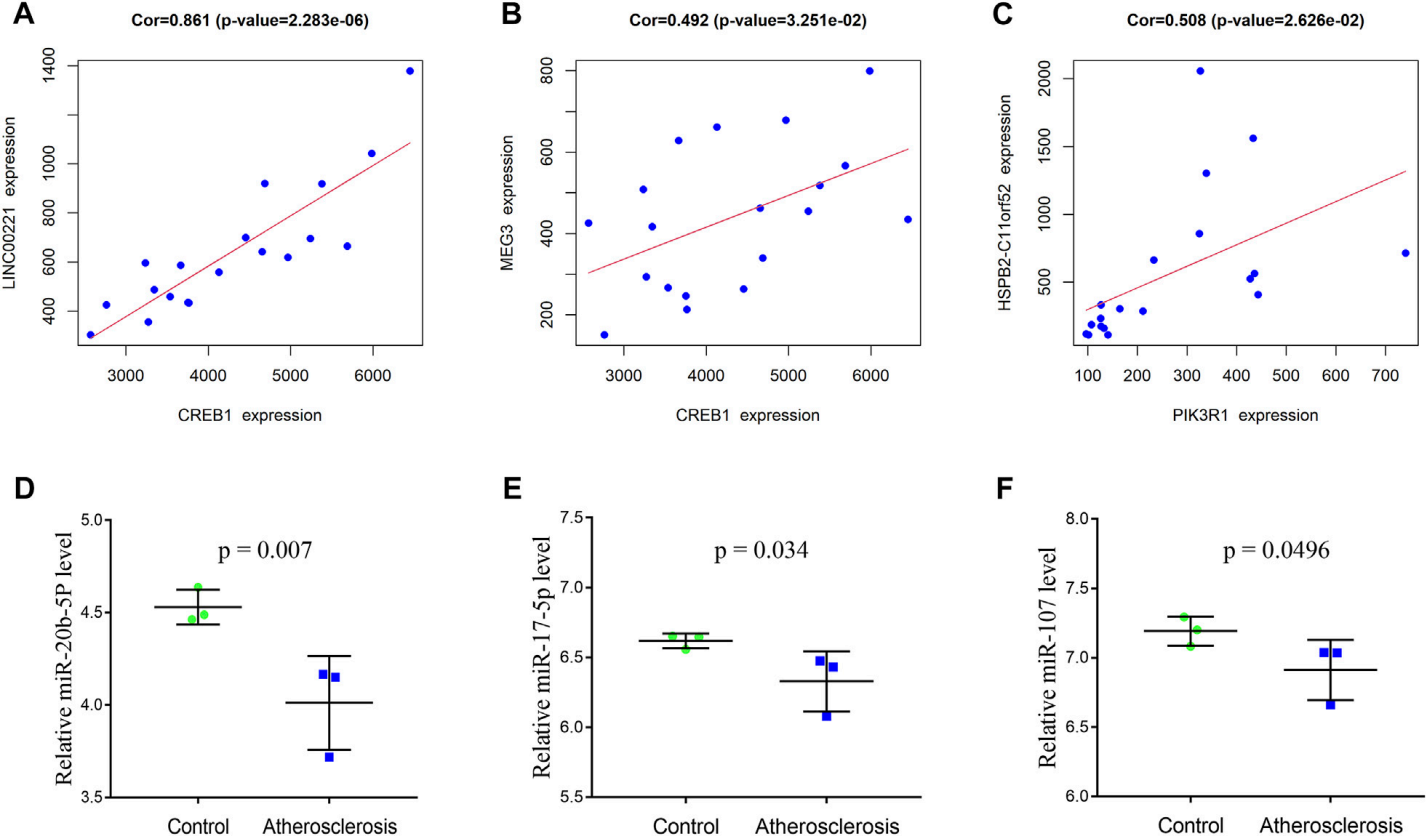
Correlation between the expression of immune-related core DE_mRNAs and their paired lncRNAs **(A**–**C)**. Validation of the miRNAs of the immune-related core ceRNA network in GSE137580 **(D**–**F)**. The results with statistically significant differences (*p* < 0.05) are shown.

To construct the immune-related core ceRNA network, the Venn method was used to analyze the intersection between lncRNA-related DE_mRNAs and immune-related genes obtained from the ImmPort database. Consequently, a total of seven genes (*TNFAIP3*, *PLXND1*, *CREB1*, *PIK3R1*, *HMGB1*, *PLAU*, and *KRAS*) were identified as lncRNA-related immune DE_mRNAs (Figure 3A). Subsequently, two genes (*PIK3R1* and *CREB1*) were identified as immune-related core genes by overlapping the lncRNA-related immune DE_mRNAs and the seven hub genes acquired in Cytoscape software. After that, immune-related core DE_mRNAs and their paired miRNAs and lncRNAs were chosen to develop the ceRNA regulatory network. In total, the immune-related core ceRNA network contained eight lncRNAs, three miRNAs, and two mRNAs (Figure 3C). Then, the correlation between the expression of immune-related core DE_mRNAs and their paired lncRNAs was analyzed. The results illustrated that the expression of *CREB1* was positively correlated with *LINC00221* (*R* = 0.861, *p* < 0.001) and *MEG3* (*R* = 0.492, *p* = 0.033) (Figures 6A,B). Similarly, *PIK3R1* was positively correlated with that of *HSPB2-C11orf52* (*R* = 0.508, *p* = 0.026), (Figure 6C). Additionally, the relative expression levels of miRNAs in the potential immune-related core ceRNA network were validated in the GSE137580 dataset and visualized by GraphPad Prism 7. As shown in Figures 6D–F, compared with the negative control group, the relative expression levels of *miR-107*, *miR-20b-5p*, and *miR-17-5p* were all low in the atherosclerosis group, due to the expression trend of *miR-20b-5p* and *miR-17-5p* being consistent with that of prediction, while that of *miR-107* was opposite to that of prediction. Moreover, the correlation ship between the expression of *LINC00221* and *CREB1* was very close. Hence, the final potential immune-related core ceRNA network in this study contained one lncRNA (*LINC00221*), two miRNAs (*miR-20b-5p* and *miR-17-5p*), and one mRNA (*CREB1*) in Table 2.

### Composition of infiltrating immune cells

**FIGURE 7:**
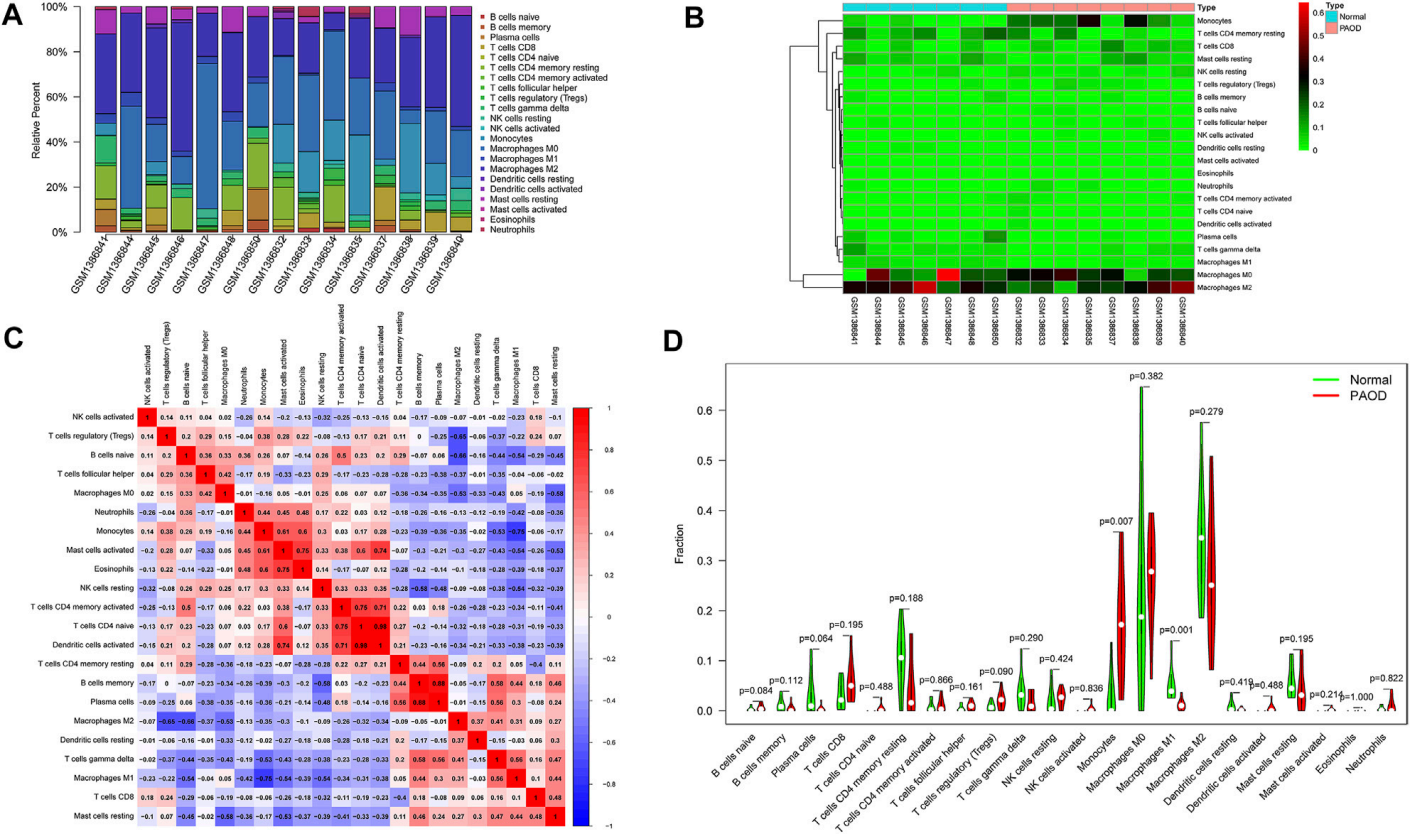
Composition of infiltrating immune cells assessed using the CIBERSORT algorithm in PAOD tissues. **(A)** Distribution of immune cell infiltration in each sample. **(B)** Heatmap of immune cell types. **(C)** Correlation among infiltrating immune cells. **(D)** Violin plot of infiltrating immune cells.

The composition of 22 infiltrating immune cells in atherosclerosis tissues of PAOD patients and normal controls was estimated using the CIBERSORT algorithm (Figures 7A,B). Since the output *p*-values of GSM1386842, GSM1386843, GSM1386849, and GSE1386836 were greater than 0.05, they were excluded for further analysis. The distribution of 22 immune cell types in each sample varied significantly, among which M2 macrophages accounted for the largest proportion. The relationships among 22 immune cells are presented in Figure 7C. Monocytes were negatively correlated with M1 macrophages (*R* = −0.75). Activated mast cells were positively correlated with eosinophils (*R* = 0.75) and activated dendritic cells (*R* = 0.74). Activated memory CD4 T cells were positively correlated with naive CD4 T cells (*R* = 0.75) and activated dendritic cells (*R* = 0.71). Naive CD4 T cells were positively correlated with activated dendritic cells (*R* = 0.98). Memory B cells were positively correlated with plasma cells (*R* = 0.88). Other immune cell subpopulations were weakly to moderately correlated. The violin plot of the immune cell infiltration difference showed that, compared with the normal control sample, two types of immune cells, monocytes and M1 macrophages, were differentially expressed. Monocytes were upregulated, while M1 macrophages were downregulated in atherosclerosis tissues of PAOD patients (Figure 7D).

### Biological experiments

**FIGURE 8:**
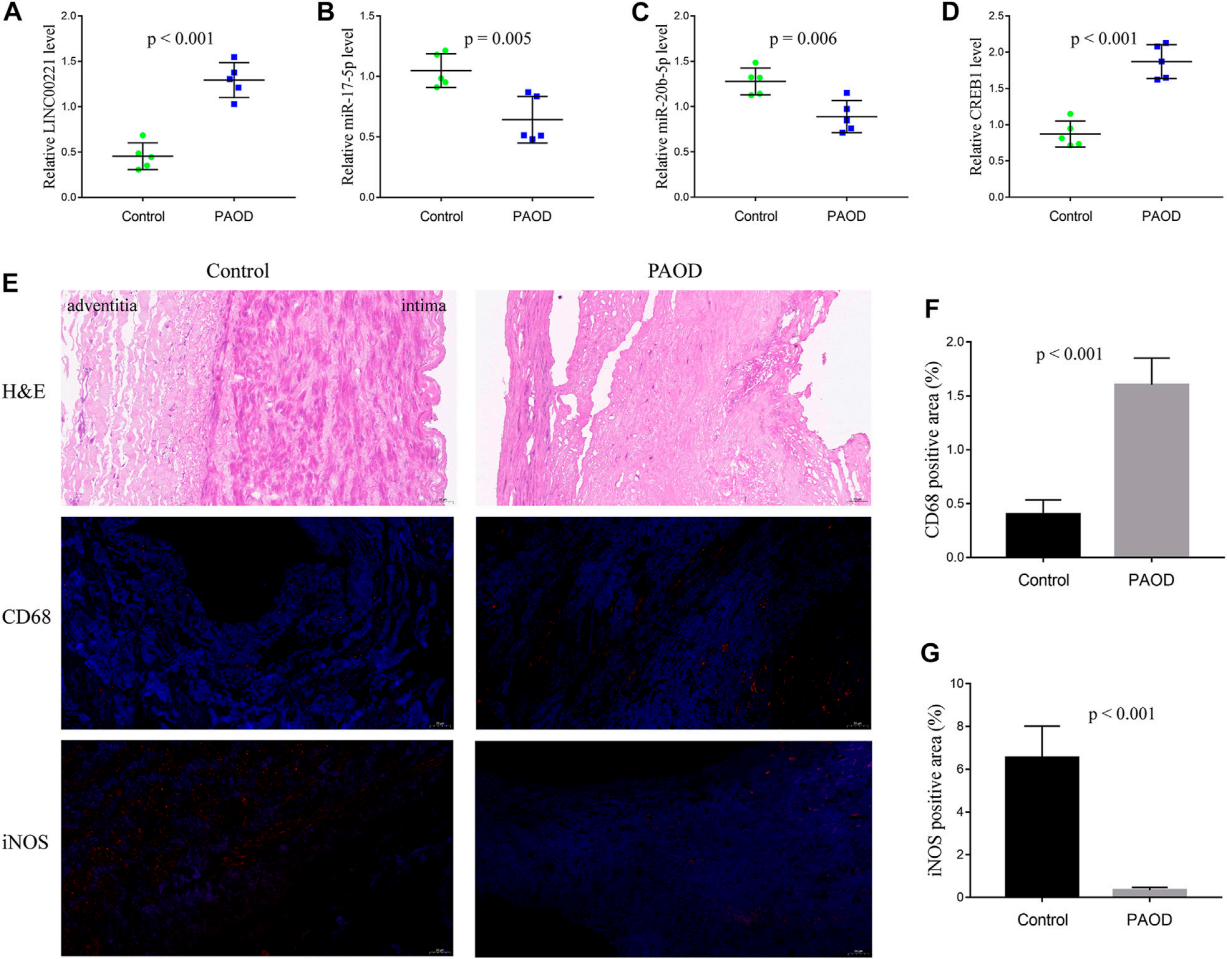
RT-qPCR validation of the immune-related core ceRNA network and evaluation of monocytes and M1 macrophages in tissue samples. Quantification of the relative expression levels of *LINC00221* **(A)**, *miR-17-5p* **(B)**, *miR-20b-5p* **(C)**, and *CREB1* **(D)** using RT-qPCR. **(E)** Representative pictures of H&E and immunofluorescence staining (magnification, ×20). Quantification of molecules in CD68 (monocyte) Fig: **(F)** and iNOS (M1 macrophage) Fig: **(G)** positive areas.

To validate the immune-related core ceRNA network in PAOD, RT-qPCR was used to detect the expression levels of the core genes. As it is shown in Figures 8A–D, compared with the normal control group, the expression of *LINC00221* and *CREB1* in the PAOD group was increased, while the expression of *miR-20b-5p* and *miR-17-5p* was decreased (all *p*-values < 0.05). Intimal structure disorder, lipid infiltration, and smooth muscle cell atrophy after endarterectomy of the femoral artery are found in Figure 8E in comparison with normal arterial intima. Subsequently, immunofluorescence staining of monocyte- and macrophage-associated molecules CD68 (monocytes) and iNOS (M1) showed that monocyte and M1 macrophage infiltration were significantly increased and decreased, respectively, in the PAOD group (all *p*-values < 0.05). The results of positive area analysis of CD68 and iNOS are shown in Figures 8F,G. These results are consistent with the bioinformatics results we predicted.

### Correlation analysis

**FIGURE 9:**
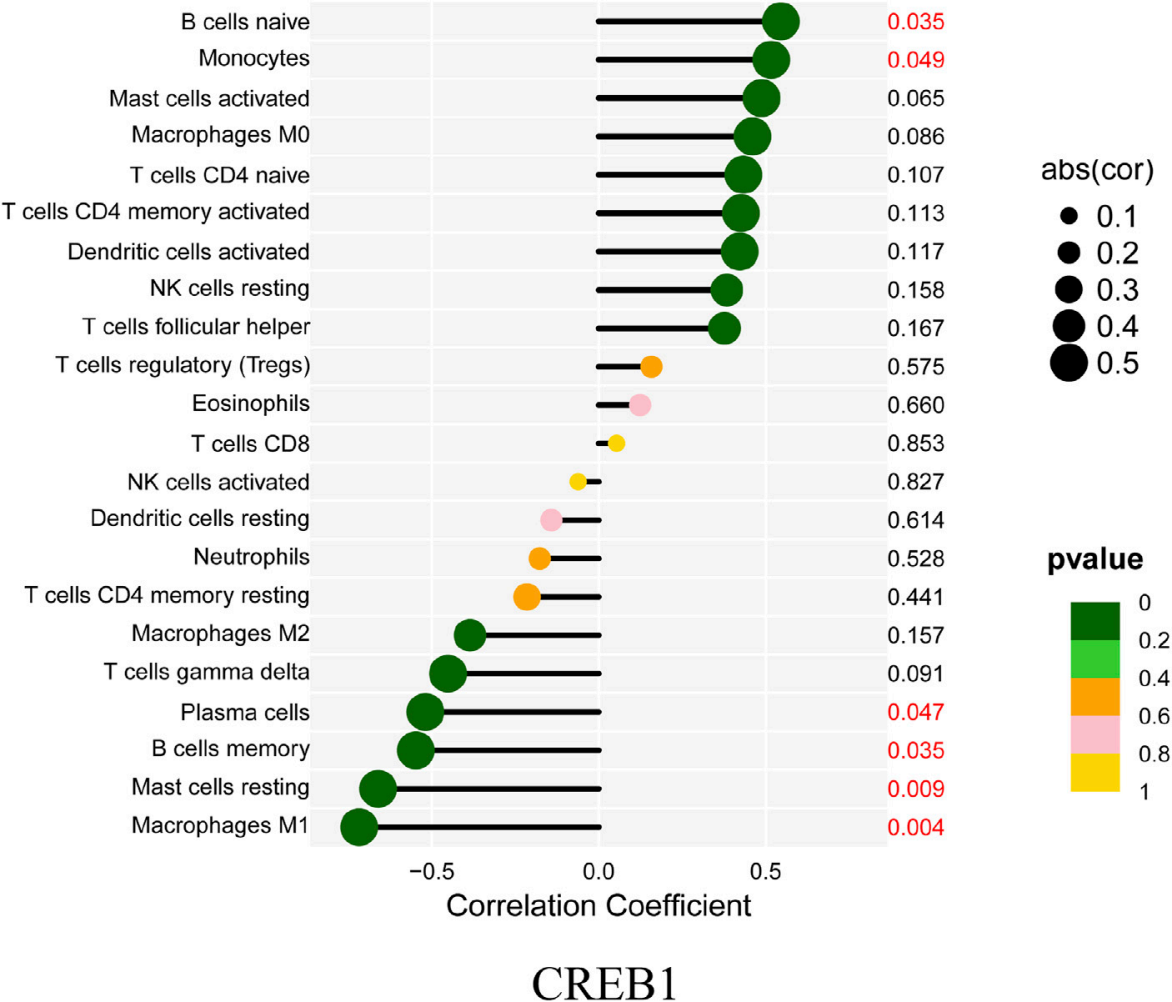
Correlation between CREB1 and infiltrating immune cells. The size of each dot is positively correlated with the strength of the correlation between genes and immune cells.

Finally, the correlation between *CREB1* and infiltrating immune cells was estimated. In this analysis, the Wilcoxon test was adopted and significantly correlated pairs with a *p*-value < 0.05. As shown in Figure 9, *CREB1* was positively correlated with naive B cells (*R* = 0.55, *p* = 0.035) and monocytes (*R* = 0.52, *p* = 0.049) and negatively correlated with M1 macrophages (*R* = −0.72, *p* = 0.004), resting mast cells (*R* = −0.66, *p* = 0.009), memory B cells (*R* = −0.55, *p* = 0.035), and plasma cells (*R* = −0.52, *p* = 0.047).

## Discussion

PAOD is a peripheral artery disease increasing with age and often leads to distal limb ischemia that results in reduced quality of life and death. Despite improvements in surgical, interventional, and pharmacological therapy of PAOD, the progression of atherosclerosis is still not prevented or curbed efficiently. Hence, in order to improve the treatment of PAOD, it is of vital significance to explore the underlying molecular mechanisms. For the past few years, the hypothesis of the ceRNA network has greatly raised the interest of researchers, and it makes the link between ncRNAs and mRNAs. On the basis of the ceRNA hypothesis, lncRNAs can act as miRNA sponges to regulate the expression of mRNAs. In this study, for exploring the potential pathogenesis of PAOD, we constructed the immune-related ceRNA regulatory network of PAOD based on the GSE57691 microarray dataset. After comprehensive analysis and careful verification, the final immune-related core ceRNA network, including one lncRNA (*LINC00221*), two miRNAs (*miR-17-5p* and *miR-20b-5p*), and one mRNA (*CREB1*), was identified. The interaction between *LINC00221*, *miR-17-5p*, and *miR-20b-5p* may regulate peripheral atherosclerosis *via CREB1*.

As post-transcriptional regulators, miRNAs play an important role in influencing the expression of downstream target genes, which are involved in a variety of physiological and pathological processes (Valencia-Sanchez et al., 2006; Bartel, 2009). During recent years, the dysregulation of miRNA expression has been found to be associated with the pathogenesis and progression of many diseases, including atherosclerosis. In fact, several miRNAs have been found to be associated with the pathogenesis of atherosclerosis. Tan et al. (2019) demonstrated that inhibiting the expression of *miR-17-5p* can suppress inflammation and reduce lipid accumulation in atherosclerosis. An et al. (2019) reported that the expression of *miR-17-5p* is significantly decreased, and lncRNA *SNHG16* can promote proliferation and inflammatory response of macrophages through the *miR-17-5p*/NF-κB signaling pathway in patients with atherosclerosis. Shen et al. (2019) identified that the expression of *circRNA0044073* was upregulated, and the expression of *miR-107* was downregulated in atherosclerotic blood cells. Moreover, *circRNA-0044073* can suppress the levels of *miR-107 via* a sponge mechanism and increase the proliferation and invasion of cells in atherosclerosis. As for *miR-20b-5p*, the reports related to its role are mainly in various cancers, but there are few reports on atherosclerosis. More research studies are needed on the role of *miR-20b-5p* in atherosclerosis in the future.

The *CREB1* gene encodes cyclic-AMP response-binding protein 1, which is a transcription factor that is a member of the leucine zipper family of DNA-binding proteins. *CREB1* has been shown to be involved in both positive and negative regulation of atherosclerosis. On the one hand, *CREB1* upregulation is observed in the vessels isolated from normal mice compared to atherosclerotic mice (Schauer et al., 2010). On the other hand, *CREB1* can activate the pro-inflammatory cytokine IL-17 that is directly responsible for macrophage accumulation and the ensuing inflammation in the atherosclerotic plaque in mice (Kotla et al., 2013). Being equally ambiguous is the role of *CREB1* in the endothelial dynamic balance. A large body of data seems to suggest that the deletion of *CREB1* in endothelial cells may result in an enhanced inflammatory response and barrier dysfunction (Chava et al., 2012; Xiong et al., 2020). On the contrary, *CREB1* can promote leukocyte adhesion by directly binding to human umbilical vein endothelial cell *ICAM1* and activating its transcription (Hadad et al., 2011). Similarly, *CREB1* interacts with *BAF47* (*BRG1*-associated factor 47) and recruits *BAF47* to the proximal neogenin 1 promoter, leading to neogenin 1 trans-activation that contributes to endothelial dysfunction (Li et al., 2022). These discrepant roles of *CREB1* may allude to its coupling to various signaling pathways targeting either the stimulation or suppression of progression in atherosclerosis. Spatiotemporally controlled *CREB1* transgenic animal models should be employed in future studies for delineating the implied role of *CREB1* in atherosclerosis.

Additionally, in this study, we described the infiltrating immune cells in peripheral arterial plaques, analyzed the differences in the abundance of immune cells between the groups, and estimated the correlation between the immune-related core genes (*CREB1*) and infiltrating immune cells. The results demonstrated that monocytes and macrophages were the main immune cell subpopulations in atherosclerosis of peripheral artery tissues. In addition, atherosclerotic plaques had increased infiltration of monocytes while having decreased proportions of M1 macrophages compared to normal artery tissues. The result that M1 macrophages are found at higher levels in normal tissues appears to be in contrast with the current knowledge of proatherogenic cells. The immune cell number is a relative percentage among each group. Atherosclerotic tissues have more immune cell infiltration, so there is a relatively lower content of M1 macrophages in these tissues than in the control tissues. Owing to an increased infiltration of immune cells, the diminution in percentage may not manifest a decreased number of M1 macrophages. If the infiltration score is readjusted, the absolute percentage of M1 macrophages in the PAOD group may not be lower than that in the normal group.

Monocytes are derived from bone marrow-derived progenitor cells, and the early stage of monocytes development may be regulated by the content of cellular cholesterol in a manner that can affect atherogenesis. Recent insight suggests that the key initiating step of incipient atherogenesis in human and animal models indicates subendothelial accumulation of apolipoprotein B-containing lipoproteins (apoB-LPs) (Williams and Tabas, 1995). The pivotal early inflammatory response to accumulated apoB-LPs is the activation of surficial endothelial cells, which leads to the recruitment of circulating monocytes (Mestas and Ley, 2008). Activated endothelial cells secrete chemokines and interact with cognate chemokine receptors on monocytes in a manner that promotes monocytes’ migration into the intima, where they differentiate into macrophages and phagocytize lipoproteins, leading to foam cell formation. Importantly, atherogenesis can be prevented or retarded in mouse models of atherosclerosis through preventing monocyte infiltration by blocking chemokines or their receptors (Mestas and Ley, 2008). The functions of macrophages within plaques are shaped largely by external stimuli such as intracellular energy metabolism (Van den Bossche et al., 2017), gut microbiota metabolites (Wang et al., 2011), and genetic and epigenetic factors including ncRNAs (Erbilgin et al., 2013; Amit et al., 2016). Traditionally, macrophages are classified into pro-inflammatory M1 macrophages (activated by lipopolysaccharide and interferon-γ) and anti-inflammatory M2 macrophages (induced by interleukin-4 and interleukin-13) (Johnson and Newby, 2009; Rath et al., 2014). In general, M1 macrophages perform processes that promote atherosclerosis progression, whereas M2 macrophages carry out functions that can restrain plaque progression and facilitate plaque regression (Peled and Fisher, 2014). M1 macrophages, through secreting cytokines, proteases, and other factors, increase the cellular expansion of lesions and cause changes in plaque morphology that result in plaque rupture and acute lumenal thrombosis. Two key changes in plaque morphology promoted by M1 macrophages are plaque necrosis and fibrous cap thinning. A previous study has confirmed that M1 macrophages can secrete matrix metalloproteinases (MMPs), such as *MMP9* and *MMP2*, which may contribute to plaque rupture, and another study shows that MMPs co-localize with M1 macrophages in advanced plaques (Huang et al., 2012). In this study, we found that the expression of the *CREB1* gene was positively correlated with monocytes (*R* = 0.52, *p* = 0.049) and negatively correlated with M1 macrophages (*R* = −0.72, *p* = 0.004). Potentially, the crosstalk between the ceRNA network and the infiltration of immune cells plays a crucial part in regulating atherosclerosis progression. Further research studies are required to clarify the complex interactions between these genes and immune cells.

However, this article has some limitations. First, only one dataset (GSE57691) that is from the west and lacks ethnic diversity was used as a data source. Moreover, the analysis of immune cell infiltration was based on the CIBERSORT algorithm, the immune cell types of which were not comprehensive. Finally, the underlying regulatory mechanisms of the ceRNA network and immune cells were not elucidated clear enough, and more functional biological experiments with larger sample sizes are needed to further verify this in the future.

## Conclusion

Taken together, in this study, the immune-related ceRNA network, by the composition of one lncRNA (*LINC00221*), two miRNAs (*miR-17-5p* and *miR-20b-5p*), and one mRNA (*CREB1*), was first constructed in PAOD. Afterward, the immune cell infiltration analysis was performed to estimate the abundance and differences of different immune cells. The final results show that monocytes and M1 macrophages were considered to be important immune cells associated with PAOD formation. Moreover, the expression of the *CREB1* gene was positively correlated with monocytes (*R* = 0.52, *p* = 0.049) and negatively correlated with M1 macrophages (*R* = −0.72, *p* = 0.004). These findings provide new insights into the pathogenesis and progression of PAOD and novel potential therapeutic targets. Perhaps in the future, new drugs can be developed for these novel potential therapeutic targets to delay the progression of PAOD and improve the long-term patency rate of vascular lumen in PAOD patients undergoing surgery or interventional therapy.

## Data Availability

The original contributions presented in the study are included in the article/Supplementary Material; further inquiries can be directed to the corresponding author.

## Glossary

**AAA**: abdominal aortic aneurysm
**apoB-LPs**: apolipoprotein B-containing lipoproteins
**BP**: biological process
**CC**: cellular component
**CD**: cluster of differentiation
**ceRNA**: competing endogenous RNA
**CREB1**: cAMP-responsive element-binding protein 1
**DCs**: dendritic cells
**DE**: differentially expressed
**FC**: fold change
**FDR**: false discovery rate
**FOXO1**: forkhead box O1
**FOXO3**: forkhead box O3
**GEO**: Gene Expression Omnibus
**GO**: gene ontology
**HAEC**: human aortic endothelial cell
**HMGB1**: high mobility group box 1
**IL**: interleukin
**IMMPORT**: Immunology Database and Analysis Portal
**iNOS**: inducible nitric oxide synthase
**KEGG**: Kyoto Encyclopedia of Genes and Genomes
**KRAS**: KRAS proto-oncogene GTPase
**LDL**: low-density lipoprotein
**LIMMA**: linear models for microarray data
**lncRNA**: long noncoding RNA
**MCC**: maximal clique centrality
**MEG3**: maternally expressed 3
**MF**: molecular function
**miRNA**: microRNA
**MMPs**: matrix metalloproteinases
**MNC**: maximum neighborhood component
**mRNA**: messenger RNA
**ncRNA**: noncoding RNA
**NK**: natural killer
**PAOD**: peripheral arterial occlusive disease
**PIK3R1**: phosphoinositide-3-kinase regulatory subunit 1
**PLAU**: plasminogen activator urokinase
**PLXND1**: plexin D1
**PPI**: protein–protein interaction
**PTEN**: phosphatase and tensin homolog
RNA: ribonucleic acid
**RT-qPCR**: real-time quantitative polymerase chain reaction
**STRING**: Search Tool for the Retrieval of Interacting Genes
**Th1**: T-helper type 1
**Th17**: T-helper type 17
**TNFAIP3**: TNF alpha-induced protein 3
**Treg**: regulatory T
**YWHAE**: tyrosine 3-monooxygenase/tryptophan 5-monooxygenase activation protein epsilon
**YWHAH**: tyrosine 3-monooxygenase/tryptophan 5-monooxygenase activation protein eta
