# Supplementary material for: A construction and comprehensive analysis of the immune-related core ceRNA network and infiltrating immune cells in peripheral arterial occlusive disease
Source: Front Genet. 2022 Sep 15;13:951537. doi: 10.3389/fgene.2022.951537 (PMC9521039; doi:10.3389/fgene.2022.951537)
Supplement: Supplementary file 1 [file Table1.DOCX]

| Table S1. Sequence of primers used for RT-qPCR. | | | |
| --- | --- | --- | --- |
| Gene |  | | Sequence (5’ to 3’) |
| LINC00221 | Forward | CCTTTATGTGGTACAGGGTTGG | |
|  | Reverse | TCGTGGTGGTTGAAAGCCC | |
| hsa-miR-20b-5p | Forward | CCTAGTAGTGCCAAAGTGCT | |
|  | Reverse | CCAGGAGTACTAGAAGTGATCA | |
| hsa-miR-17-5p | Forward | TGCGCCAAAGTGCTTACAGTGCA | |
|  | Reverse | CCAGTGCAGGGTCCGAGGTATT | |
| U6 | Forward | GCTTCGGCAGCACATATACTAAAAT | |
|  | Reverse | CGCTTCACGAATTTGCGTGTCAT | |
| CREB1 | Forward | ATTCACAGGAGTCAGTGGATAGT | |
|  | Reverse | CACCGTTACAGTGGTGATGG | |
| GAPDH | Forward | GATTTGGTCGTATTGGGCGC | |
|  | Reverse | TTCCCGTTCTCAGCCTTGAC | |
